# Helical unwinding and side-chain unlocking unravel the outward open conformation of the melibiose transporter

**DOI:** 10.1038/srep33776

**Published:** 2016-09-23

**Authors:** Li-Ying Wang, Vidhya M. Ravi, Gérard Leblanc, Esteve Padrós, Josep Cladera, Alex Perálvarez-Marín

**Affiliations:** 1 Unitat de Biofísica, Departament de Bioquímica i de Biologia Molecular, Facultat de Medicina, and Centre d’Estudis en Biofísica, Universitat Autònoma de Barcelona, 08193 Bellaterra, Barcelona, Spain; 2 Direction des Sciences du Vivant, Direction des programmes et valorization, CEA Fontenay-aux-Roses, 92265 Fontenay-aux-Roses CEDEX France

## Abstract

Molecular dynamics simulations have been used to study the alternate access mechanism of the melibiose transporter from *Escherichia coli*. Starting from the outward-facing partially occluded form, 2 out of 12 simulations produced an outward full open form and one partially open, whereas the rest yielded fully or partially occluded forms. The shape of the outward-open form resembles other outward-open conformations of secondary transporters. During the transporter opening, conformational changes in some loops are followed by changes in the periplasm region of transmembrane helix 7. Helical curvature relaxation and unlocking of hydrophobic and ionic locks promote the outward opening of the transporter making accessible the substrate binding site. In particular, FRET studies on mutants of conserved aromatic residues of extracellular loop 4 showed lack of substrate binding, emphasizing the importance of this loop for making crucial interactions that control the opening of the periplasmic side. This study indicates that the alternate access mechanism for the melibiose transporter fits better into a flexible gating mechanism rather than the archetypical helical rigid-body rocker-switch mechanism.

The viability of the living cell depends on the transport of materials across the cell membrane. Such a transport may be facilitated by channels or take place against a concentration gradient via ATP-dependent processes or via the dissipation of electrochemical gradients. Among the latter, known as secondary transporters, the Major Facilitator Superfamily (MFS)^1^ corresponds to a set of membrane proteins structurally organized in the membrane as 12 transmembrane helices that transport substrates by the so called alternate-access mechanism.

In the alternate-access mechanism the substrate-binding site is accessible alternatively from the cytoplasmic and from the extracellular side. These two conformations (outward and inward open) transit between the formation of two occluded states (closed to both sides)^2^. From the structural information derived from several crystallized transporters that belong the Major Facilitator Superfamily (MFS), a common fold has been described, consisting in two helical bundles (N and C-terminal domains) of six transmembrane segments each^2^,^3^. Within the two helical bundles, helices would be organized in the form of two inverted-topology repeats, each made of three consecutive helices which would be essential for the conformational swapping in the alternating mechanism^4^,^5^. Identifying hydrophobic and salt-bridge interactions that are key for the conformational changes that occur during transport is crucial in order to understand the alternate access mechanism.

High-resolution structure determination (X-ray crystallography, cryo-electron microscopy, etc.) is nowadays the most powerful way to obtain atomic resolution structural information of membrane proteins. Structural biology in combination with biochemical and biophysical information, and with the data from site-mutagenesis experiments, can elucidate the molecular mechanism by which membrane proteins function. However, since high-resolution structures represent a snapshot of complex molecular processes involving different intermediate structures, the speed at which the protein dynamics is revealed by experimental methods is still slow.

Molecular dynamics (MD) allows a quick exploration of protein dynamics from the crystallographic structures. The advance on computational methods has made possible the use of powerful software in standard GPU-based IT platforms^6^,^7^,^8^,^9^,^10^,^11^,^12^,^13^, allowing the use of MD techniques in the average protein-membrane research lab. This advance represents a powerful tool to test experimental results, build new hypothesis, and carry out iterative *in vitro*/*in silico* approaches, which permit the study of key transitions between metastable states in membrane protein dynamics^14^,^15^,^16^,^17^. Enhanced MD techniques^18^,^19^ in combination with GPU-clusters could be used to explore the full transport cycle for a transporter, which is inaccessible in terms of computational power and time scale for a single-GPU workstation using all atom MD.

The melibiose permease from *Escherichia coli* (MelB) is a member of the glycoside-pentoside-hexuronide cation symporter family of MFS that catalyzes the active transport of disaccharide melibiose using an electrochemical gradient of either Na^+^, Li^+^ or H^+^ as a co-substrate^20^,^21^. The crystal structure of an outward-open partially occluded form of the MelB of *Salmonella typhimurium*, (MelB_St_) reveals a typical MFS fold consisting of 12 transmembrane helices^22^. This structure provides the first static snapshot of MelB and is key to understand and study the transport mechanism at atomic level. In previous studies, some side chains such as D19, D55, D59, D124, R149 or K377 have been proposed to outline the substrates’ binding site^22^,^23^,^24^,^25^,^26^. In particular, the crystal structure verifies that these side chains are located in the inner protein space. Therefore, it is of major interest to elucidate the conformational changes by which this space becomes exposed to the bulk region to continue the transport cycle by binding the co-substrates, Na^+^ and melibiose. In the present study, we use Ethayathulla *et al*.’s^22^ outward partially occluded structure as a template to study the alternate access transport mechanism of MelB using MD. The results reveal an outward fully open conformation. Changes in the curvature of TM7, the role of a periplasmic hydrophobic lock and the conformational dynamics of several ionic locks are discussed, together with the significance of the results with respect to other MD studies of similar transporters and the implications in relation to the transport mechanism model.

## Results and Discussion

**Figure 1:**
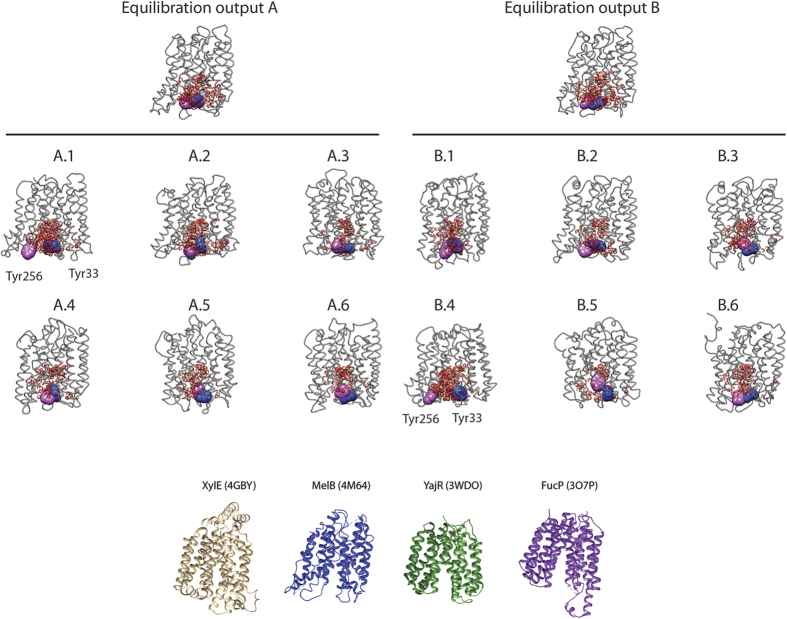
Replica analysis. Comparison of the 12 replicas originated from two different equilibration outputs. The state represented corresponds to the 100 ns step. The periplasmic opening is indicated by the red Van der Waals surface plot of the waters used to calculate volumes in Table 1. Y33 and Y256 are indicated as large spheres in blue and purple, respectively. The crystal structures of XylE, MelB, YajR and FucP are plotted for comparison. Replica A.1 embedded in the lipid bilayer, solvated and ionized is depicted in Fig. S3.

In this work, we use MD to get further insight into the conformational changes in the alternating access transport mechanism of MelB from *Escherichia coli*. We have built a protein atomistic model for *E. coli* MelB using the tridimensional coordinates of MelB_St_ structure^22^, which is in an outward partially occluded state. These orthologs share an 85% sequence identity and all essential amino acids are conserved (Fig. S1). For MD simulations we have embedded MelB in a 1-Palmitoyl-2-oleoyl-sn-glycero-3-phosphoethanolamine (POPE) lipid bilayer under 150 mM NaCl ionic strength and solvated it with TIP3P waters^27^ using the CHARMM force field^11^,^12^. Taking the substrate-free outward partially occluded state as the starting structure, we generated two different equilibration runs (Eq(A) and Eq(B), see methods for details) to run a total of 12 full-atom MD simulations of 100 ns each (Fig. 1 and Fig. S2), in the absence of melibiose. Nine replicas yielded a partially occluded-like state or even a totally occluded state as judged by (i) the plot of distances between the Cγ of Y33 (extracellular end of TM1) and Y256 (extracellular end of TM7), compared to the initial state (post-equilibration), and (ii) the water occupancy in the periplasmic cavity measured using Volarea^28^ (Table 1). This double criteria approach allowed us to discriminate between replicas simply increasing its inner cavity pore radius compared to replicas opening towards the periplasmic side. According to our MD replicas, conformational changes leading to the transporter opening are under-populated (<25%) in agreement with previous reports for other transporters^29^. The replicas A.1 and B.4 show the Y33–Y256 largest distances (Fig. 1 and Table 1), reaching a substrate-free outward open state. Replicas A.4, A.6 and B.3 show a decrease in the distance between the Cγ of Y33 and Y256 suggesting a further closing of MelB from the partially occluded state. Replicas A.1, B.4 show the largest water cluster located at the extracellular side, indicating opening of the periplasmic space (Fig. 1 and Table 1) whereas most of the replicas show a decrease in the water occupancy of the periplasmic cavity (Table 1). Replica A.2 shows parameters slightly different from the starting Eq(A), such as larger Y33–Y256 distance and periplasmic water volume, indicating a partially open state.

### Conformational changes in helices and loops upon outward opening

**Figure 2:**
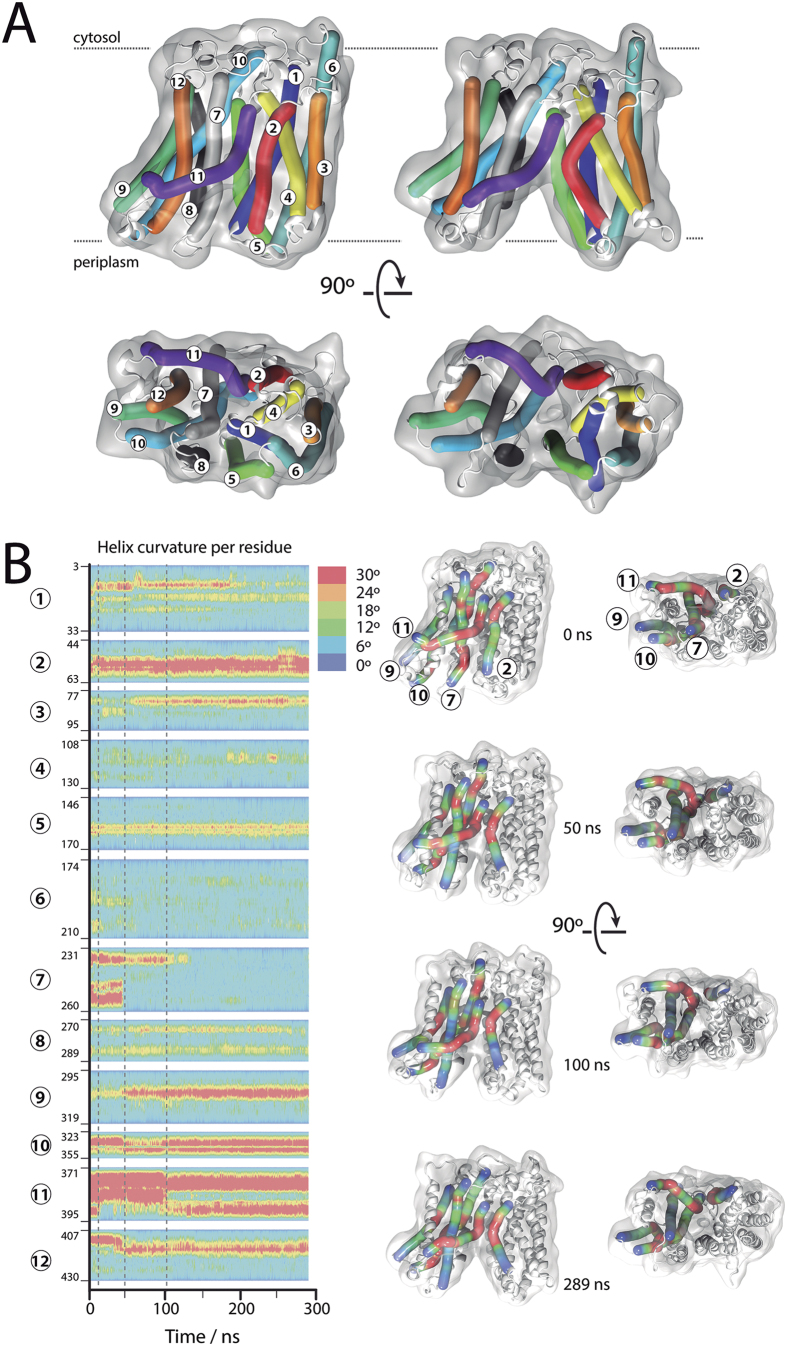
(**A**) Bendix plot^13^ rendered in VMD^9^ representing the transition from a partially occluded to an outward full open state. (**B**) Heat map of helices curvature per residue as a function of time. Scale bar indicates the helix curvature degree. Grey dashed lines highlights time points for 3D snapshots at 0, 50, 100, and 289 ns shown on the right side.

The MD for A.1 replica was extended up to 289 ns (Fig. 2 and Supplementary movie), since this replica showed the largest periplasmic cavity volume. The transition from the substrate-free outward partially occluded to full open state is characterized by dramatic conformational changes (Fig. 2A). The simulations reveal that the first conformational changes occur in the cytoplasmic loop CL5 and the extracellular loops EL1, EL2 and EL4 (Fig. S4). This is followed by a helix unwinding effect in the extracellular segment of TM7 as shown by the decrease of the helix curvature at around 50 ns (Fig. 2B). The structural distortion decreases from G252 towards the cytoplasmic side, resulting in a more regular helix (Fig. S5). In parallel, changes in the degree of curvature of other helices such us TM9 and TM12 are observed (Fig. 2B). This early set of helical relaxation/torsion events results in a considerable separation of TM7 from the N-terminal helical bundle and drives the opening of the extracellular region. The position of TM7 with respect to TM1 and TM5 in the partially occluded and open conformations (Fig. 2A) shows a remarkable coincidence with the relative positions of these helices in the reported outward-open partially occluded structure of XylE^30^ and the outward-open structure of FucP^31^ (Fig. S6). At around 100 ns, the protein shows a full open conformation that hardly changes until the end of the simulation (Figs 2B and S2). The asymmetric MelB arrangement defined in two helical bundles varies from an “order parameter” (the shortest distance between Cα atoms of TM1 and TM7 defined by Stelzl *et al*.^32^) of about 9 Å (T34–Y256) to 24 Å in going from the partially occluded to full open states. In the cytoplasmic gate, the order parameter varies from 9.5 Å (Y128 in TM4–V346 in TM10) to 10.5 Å. These values fit very well with the values found by Stelzl *et al*.^32^ as defining the occluded and outward-open states, respectively, for MFS transporters.

### Ionic and hydrophobic interactions change upon opening

**Figure 3:**
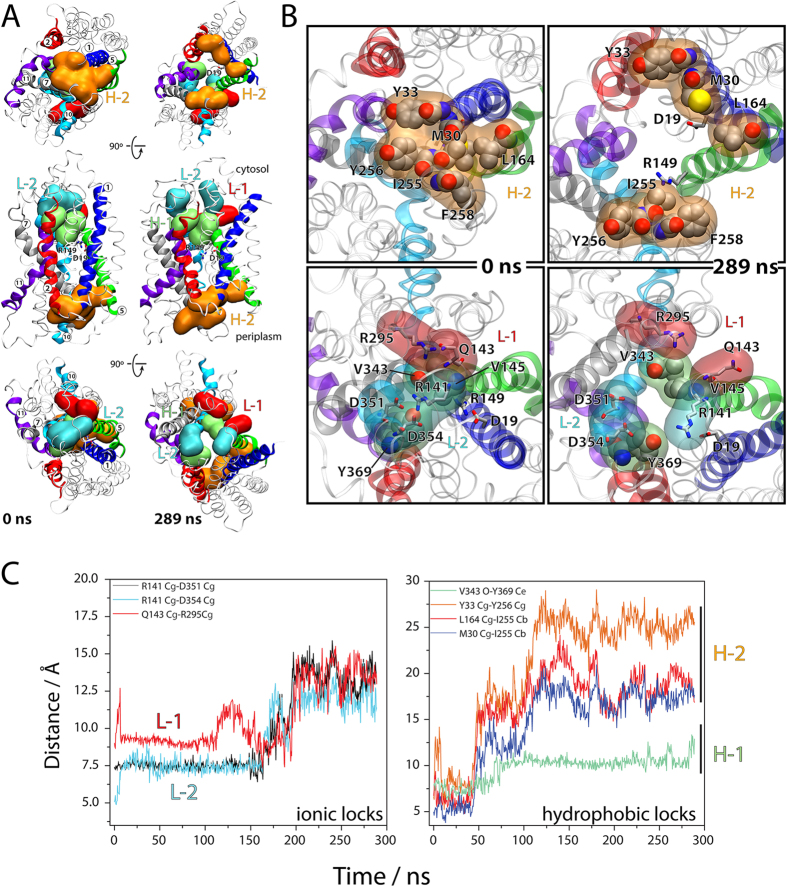
(**A**) Illustrative representation highlighting the hydrophobic H-1 (V145, V343, Y369) and H-2 (M30, Y33, L164, I255, Y256, F258), and ionic L-1 (Q143, R295) and L-2 (R141, D351, D354) locks. (**B**) Details of the partially occluded state (0 ns) and the outward full open state (289 ns). (**C)** Interatomic distances as a function of time for specific residues in hydrophobic and ionic locks.

The *S. typhimurium* outward partially occluded state of MelB contains a set of ionic locks and hydrophobic patches^22^. We have mapped these interactions in our system (Fig. 3). The ionic locks (L-1 to L-3) define a set of electrostatic interdomain interacting residues stabilizing a defined conformation (L-1 and L-2 shown in Fig. 3A,B in red and cyan surfaces, respectively). Residues such as L131, L144, V145, V343, I347, and Y369, form an interdomain hydrophobic lock H-1 (in light green in Fig. 3A,B). MelB opening destabilizes all three cytoplasmic locks, and therefore the interdomain interactions between R141 and D351–D354, between Q143 and R295, and between R363 and G74, respectively, are broken (Fig. 3B,C). The cytoplasmic hydrophobic patch is also broken, specifically, by the fluctuation of the cytoplasmic ends of TM4, TM5, TM10 and TM11 that pull L131, L144, V145, and Y369 apart from V343 and I347 (Fig. 3B,C).

Our analysis of the partially occluded structure shows interdomain hydrophobic locks in the extracellular side that hold the N and C domains together. The hydrophobic cluster formed by the conserved residues I255, Y256 and F258 at the end of TM7 form a hydrophobic lock H-2 with M30 and Y33 in TM1 and with L164 in TM5 (Fig. 3A,B). The interactions between TM1-TM5 and TM7 are lost in the outward-open state (Fig. 3B,C) while they are tighter in the fully occluded state (Fig. S7). This is consistent with the presence of an extracellular gate between TM1 and TM7 for the *Geobacillus kaustophilus* H^+^-driven peptide symporter GkPOT^3^.

### The EL4 loop and the scissor’s model

**Figure 4:**
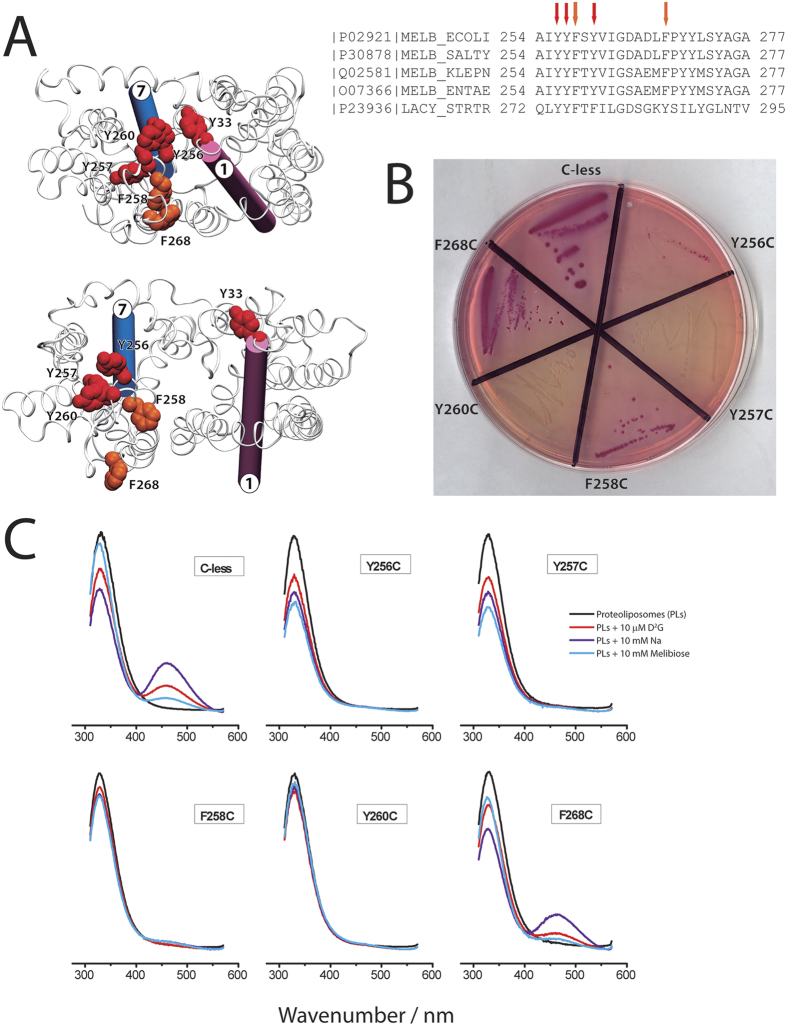
(*A*) Conserved aromatic residues in melibiose permease in the extracellular end of TM7 (EL4). Alignment includes MelB proteins for *E. coli*, *S. typhimurium*, *Klebsiella pneumoniae*, and *Enterobacter aerogenes*, and LacY for *Streptococcus thermophiles. The residues mutated to cysteine in this study are indicated by arrows.* Tyrosine residues are indicated in red and phenylalanine in orange, respectively; TM1 and TM7 are drawn in mauve and blue cylinders, respectively. (**B**) Melibiose metabolic test to measure indirectly melibiose transport (see text for details). (**C**) FRET from tryptophan side chains to D^2^G (λex = 290 nm) in nominally Na^+^-free 100 mM potassium phosphate (pH 7.0) and 100 mM KCl. Emission fluorescence was recorded before and after the consecutive additions of 10 μM D^2^G, 10 mM NaCl, and 10 mM melibiose. Each spectrum is the average of three scans.

A recent transport model (scissor’s model) has been proposed for membrane transporters in which the periplasmic gate consists in key interactions between helices pairs TM1-TM2 and TM7-TM8 for oligopeptide transporters^5^. In our simulation, early changes in the EL4 between TM7 and TM8 trigger the unwinding effect on the periplasmic side of TM7 (Fig. 2 and Fig. S2). To assess the role of the EL4 loop in the opening of MelB we focused in the cluster of conserved aromatic residues Y256, Y257, F258, and F268 (Fig. 4A) between TM7 and TM8. Especially Y256 and Y260 are forming an aromatic girdle with another set of aromatic residues (Y31, Y32, Y33) in TM1 (Fig. 4A and Fig. S1). Single-cysteine mutants Y256C, Y257C, F258C, Y260C and F268C were constructed, expressed, and compared to the wild type-like C-less variant.

Melibiose transport in *E. coli* was tested indirectly for the different mutants using a metabolic test that monitors the acidification of the media after melibiose cleavage. Strains capable of transporting melibiose will turn purplish red after the metabolic reaction (Fig. 4B). Y257C and Y260C showed no purple color, while Y256C and F258C yielded few colonies turning purple but lower compared to the number of colonies in F268C and the C-less variant. We purified the C-less and mutant proteins and after reconstitution into proteoliposomes Na^+^-dependent FRET studies were carried out. None of the mutants showed FRET signals in the presence of the fluorescent sugar analog D^2^G (Fig. 4C) except for F268C. The FRET signal was not observed after consecutive additions of Na^+^ or melibiose, except for F268C, arguing for undetectable levels of binding and transport for Y256C, Y257C, F258C, and Y260C. These results highlight the importance of the aromatic residues in EL4, between TM7 and TM8 to control the opening of the periplasmic side of MelB (Fig. S8), probably through the interaction with aromatic and non-polar residues in TM1, in agreement with the scissors model for oligopeptide transporters^5^. Although in oligopeptide transporters the periplasmic gates consisting of TM1-TM2 and TM7-TM8 consist of salt bridges, in the case of MelB it has been suggested that aromatic residues may recognize/guide the sugar to its binding site through CH-π interactions with the pyranosyl ring^22^.

### Critical interactions at the transporter’s core

**Figure 5:**
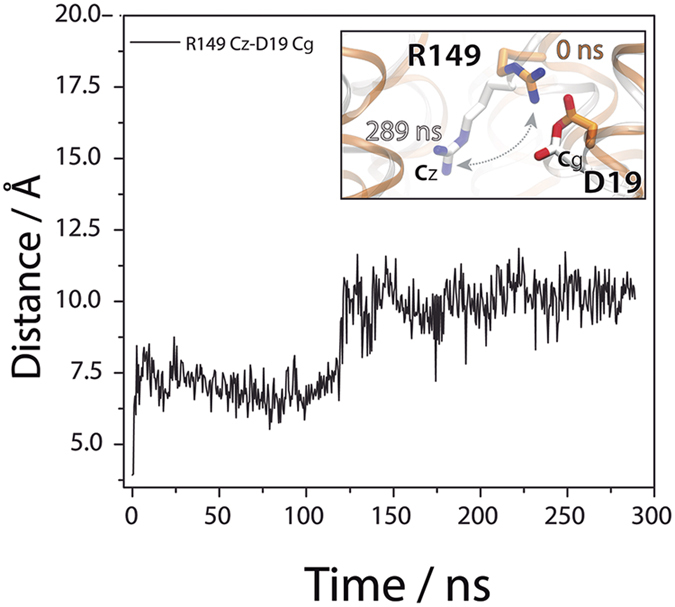
Interatomic distances between residues D19 and R149. Inset shows a superposition of the structures at 0 ns (orange) and 289 ns (white) showing the change of orientation of R149 side chain.

**Figure 6:**
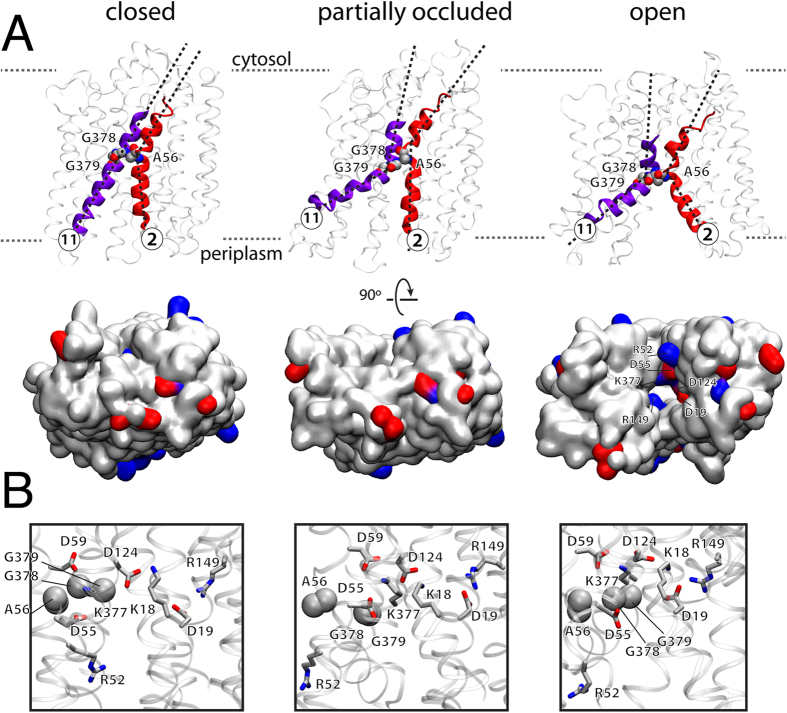
(**A**) The ribbon diagram highlights the helical torsion and orientation changes in helices TM2 and TM11 emphasizing the A56–G378–G379 knob. The bottom representation as QuickSurf in VMD depicts the exposure of the substrate binding site residues, where blue and red indicates basic and acidic residues, respectively. For the representation of the occluded state we have selected replica B.1. (**B**) Representation highlighting key residues in MelB substrate binding and transport.

In the center of the transporter, the salt bridge between D19 and R149 is broken during the opening of the structure as the distance between D19 and R149 increases due to a reorientation of R149 (Fig. 5). As previously described^33^, mutations of R149 may favor the inward-open conformation (i.e., R149C) or the outward-open conformation (R149Q). The side chain at position 149 appears to be crucial for the alternating access mechanism of MelB and the D19-R149 salt bridge could be relevant for the opening/closing mechanism. By contrast, the interdomain salt bridge between K377 and D59 remains stable during the simulation. K377 is critical for the correct configuration of the Na^+^-binding site^27^ but it is apparent that the D59–K377 interaction is also important for the correct maintenance of the overall MelB structure. Such a salt bridge in the central region of MelB linking TM2 and TM11 may be essential for holding the central region of the N and C interdomains, as may be the hydrophobic interaction established by A56 in TM2 with residues G378 and G379 in TM11 (Fig. 6A). The A56–G378–G379 knob appears as an element in MelB to accommodate the helical torsions observed when the protein goes from the occluded to the fully open state (Fig. 6A). A closer look at the area where helices 2 and 11 are adjacent (Fig. 6) reveals a cluster composed mainly of acidic and basic residues that have been recognized as important elements of the substrates binding sites (D55, D59, D124 and K377). Within this set of charged residues, R52, D55, D59, R149, and K377 can be observed in the outward fully open form of the protein lining the surface of the cavity open to the periplasm (Fig. 6). R52 may act as a gate-keeper that caps the cavity upon occlusion of the structure, and inside, D55 and D59 define the sodium binding site, surrounded by K377 and R149, which may behave as two electrostatic barriers that would lead the cation to its binding site (Fig. 6).

**Figure 7:**
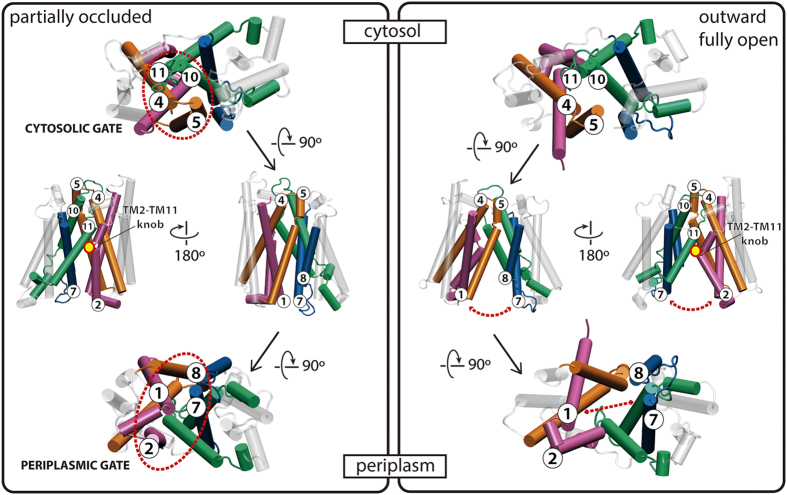
Cartoon showing MelB opening. According to the model proposed by Fowler *et al*.^5^ for oligopeptide transporters, the pairs of helices TM4–TM5 (orange) and TM10–TM11 (green) define the cytosolic gate, whereas the pairs of helices TM1–TM2 (mauve) and TM7–TM8 (blue) define the periplasmic gate; gates indicated by a dashed red ellipsoid in our MelB opening. The opening of MelB towards the periplasmic side (dashed red arrowed lines) barely affects the cytosolic gate, dividing the protein in two halves at the level of the TM2–TM11 knob (red-line yellow dot).

The alternating access mechanism common to some MFS transporters evolves from the cytoplasm-open conformation to the periplasm-open one via an occluded conformation. This transition may be important in order to avoid the quick dissipation of the proton or sodium electrochemical gradients partially responsible for the uphill transport of sugars and other substrates. Some of the crystal structures of MFS transporters, such as XylE and MelB_St_, are structural snapshots that experimentally proves the existence of such energetically favored occluded structures. Stelzl *et al*.^32^ have recently proposed that the alternating access mechanism via the formation of an occluded state would rely on the existence of two flexible gates (one on the cytoplasmic side, the other on the periplasmic one), also in agreement with the scissors’ model for oligopeptide transporters proposed by Fowler *et al*.^5^. Our MD model for MelB, shows some details that agree very well with the flexible gating mechanisms proposed by Stelzl *et al*.^32^ and the scissors’ model by Fowler *et al*.^5^. According to our MD simulation, the opening of the protein structure from the partially occluded conformation implies the unlocking of some hydrophobic interactions between TM1 and TM7 on the periplasmic side a type of interaction that coincides well with the periplasmic flexible gate that would be responsible for occluding this side of the protein. This cytoplasmic gate implicating the interaction of TM1 and TM7 (via mainly hydrophobic residues) has been described for Lac Y and other sugar transporters^32^. This cytosolic gate is represented as well in MelB (Fig. 7). The unlocking of the ionic lock on the cytoplasmic side of MelB, named as L2 by Ethayathulla *et al*.^22^, is appreciable in our MD system, fitting with the cytoplasmic gate involving residues in TM4 and TM10 described by Stelzl *et al*.^32^. In MelB, the cytoplasmic end of TM5 (instead of the cytoplasmic end of TM4) interacting with TM10 would define the cytoplasmic gate (Fig. 7). In other MFS transporters, such as the oligopeptide transporter, this cytoplasmic gate consists on the helical pairs TM4–TM5 and TM10–TM11^5^, which in our MD simulation remains obviously closed, since we are observing events in the periplasmic opening (Fig. 7). The periplasmic gate defined in the scissors’ model for oligopeptide transporters consists on the helical pairs TM1–TM2 and TM7–TM8^5^. For MelB, in this TM1–TM2 and TM7–TM8 helical pairs, there are two aromatic clusters, one in the extracellular end of TM1 and another in the extracellular end of TM7, which appear relevant for MelB in the periplasmic gate. Our site-directed mutagenesis experimental results show that modifying the TM7–EL4 aromatic cluster severely affects melibiose transport, confirming the presence of key residues in what is postulated as the periplasmic gate for oligopeptide transporters, as probably happens with MelB (Fig. 7). From our MD simulations, it appears that the TM2–TM11 knob (Fig. 7) would act as a key element connecting the cytosolic gate helical pairs (TM4–TM5 and TM10–TM11) with the periplasmic gate helical pairs (TM1–TM2 and TM7–TM8). This TM2–TM11 knob would act as a synchronizing element between events happening in the periplasmic and the cytosolic sides, or at least, during the helical unwinding in the occluded to outward open transition.

## Concluding remarks

Our results favor a mechanism by which helical flexibility would permit a more sequential opening (one opens after both have been closed) rather than alternate opening (one opens at the same time that the other is closing) of the cytoplasmic and periplasmic sides. A flexible gate-based alternating mechanism would imply a degree of helical flexibility (the occurrence of helices tilts, torsions and windings). The conformational changes we have detected for TM2, TM11, TM5 and TM7 for the molecular transition from MelB partially occluded form to an outward-open one would argue against the rigid-body motion implied by a literal rocker-switch mechanism.

MD simulations show several limitations due to different factors as the accessible time scale, the choice of a suitable force field or the correct lipid bilayer environment^18^. The present study combining experimental and computational approaches helps to understand the molecular mechanism and conformational changes involved in a discrete step of the sugar transport cycle of MelB. Future perspectives for the study of discrete conformational transitions between metastable or quasi-metastable conformations of membrane transporters in the μs-to-ms time range will combine all-atom with coarse-grained MD simulations, together with further biophysical analysis to allow researchers to get deeper insight into the molecular mechanism of transporter proteins.

## Methods

### Molecular dynamics simulations

The coordinates of molecule-A of MelB from *Salmonella typhimurium*^22^ (PDB entry 4M64) where used with the CHARMM-GUI server^7^ in order to change all divergent amino acids to those of MelB from *Escherichia coli* (a total of 60 substitutions). To remove any possible steric clash, this was followed by a short energy minimization *in vacuo* using the NAnoscale Molecular Dynamics (NAMD) program^6^. Using the CHARMM-GUI Membrane Builder^7^, this *E. coli* MelB was inserted into a bilayer of 95 × 95 Å^2^ consisting of 218 1-Palmitoyl-2-oleoyl-sn-glycero-3-phosphoethanolamine (POPE) molecules, by the replacement method. The protein orientation was calculated from the Orientations of Proteins in Membranes database^8^, by minimizing its transfer energy from water to the membrane. Using the Visual Molecular Dynamics (VMD) program (1.9.2 version)^9^ a water layer of 15 Å on top and bottom of the protein/lipid system was added, with waters also filling the free space in the protein interior (a total of 20,885 water molecules). The system was complemented with NaCl at a concentration of 0.15 M with the “Add Ions” extension of VMD (59 Na^+^ and 63 Cl^−^ per MelB). The system had a total of 97,184 atoms.

The system was energy minimized for 100,000 steps followed by equilibration for 200 ps using an integration step of 1 fs with NAMD. Equilibration was done by slowly heating the system from 0 to 298 K in steps of 2 K. Molecular Dynamics was produced with the ACEMD program^10^ running on a PC with a GPU graphic card. The CHARMM force field parameters^11^,^12^ were used for lipid and protein atoms and the TIP3P model for water. Starting from the same minimization result, two different equilibration runs were done, resulting in two different equilibration outputs, Eq(A) and Eq(B). Six different MD replicas were produced starting from Eq(A) and six more starting from Eq(B) for a minimum of 100 ns at constant pressure and temperature, using the particle mesh Ewald method full-system periodic electrostatics. A time step of 4.0 fs, rigid bonds on all hydrogen-heavy atom bonds and a hydrogen scale parameter of 4 were used to speed-up the calculations^10^. A switching distance of 8 Å, cutoff of 10 Å, electrostatic contribution every 2 steps, Langevin thermostat at 298 K and damping of 2 ps^−1^ were set. Pressure was set at 1 atm using the Berendsen barostat. Root-mean-square-deviation (RMSD) was calculated using the VMD extension “RMSD Visualizer Tool”. Helices were plotted using the Bendix program^13^ of VMD.

### MelB expression, purification, reconstitution and functional assays

MelB (C-less) and the mutants were cloned and expressed in DW2R *E. coli* cells, and purified as reported previously^34^,^35^,^36^. Mutants were checked for protein expression by immunoblotting against the His-tag fused at the C-terminus of MelB. Mutant single colonies were streaked and the melibiose metabolism of the mutants was tested in MacConkey agar plates containing melibiose as the main carbon source and neutral red as a pH indicator. Strains capable of transporting melibiose should appear purple due to the acidification of the pH induced by melibiose cleavage after being transported into the cell. MelB reconstitution into *E. coli* lipids proteoliposomes was performed as previously described^27^.

Fluorescence measurements were done at 20 °C with a QuantaMaster^TM^ spectrofluorometer and data processed with Felix 32 software (Photon Technology International). Trp fluorescence spectra were acquired by setting the excitation wavelength at 290 nm (half-bandwidth of 5 nm) and collecting the emission spectrum in 100 mM potassium phosphate buffer (pH 7.0). Na^+^-dependent FRET signals (λex, 290 nm; half-bandwidth, 5 nm) of proteoliposomes (30 μg of protein/ml) were obtained before and after the additions of 10 μM D^2^G, 10 mM NaCl, and 10 mM melibiose.

FRET from Trp side chains to D^2^G (λex = 290 nm) in nominally Na^+^-free 100 mM potassium phosphate (pH 7.0) and 100 mM KCl. Emission fluorescence was recorded before and after the consecutive additions of 10 μM D^2^G, 10 mM NaCl, and 10 mM melibiose. Each spectrum is the average of three scans.

## Additional Information

**How to cite this article**: Wang, L.-Y. *et al*. Helical unwinding and side-chain unlocking unravel the outward open conformation of the melibiose transporter. *Sci. Rep.* **6**, 33776; doi: 10.1038/srep33776 (2016).

## Supplementary Material

Supplementary Information

Supplementary Video

## Figures and Tables

**Table 1:** List of MD replicas to study the opening of the *E. coli* MelB permease.

Replica^a^	Length (ns)	Periplasmic Cavity Volume (Å^3^)^c^	Cγ_Y33_–Cγ_Y256_ Distance (Å)	OPEN
A.0	0	5,270	6.64	No
A.1	288.96^b^	7,969	17.23	Yes
A.2	100	6,030	9.04	Partially
A.3	100	3,229	7.03	No
A.4	100	3,898	4.87	No
A.5	100	2,953	7.03	No
A.6	100	3,664	6.40	No
B.0	0	5,230	6.85	No
B.1	100	4,555	6.87	No
B.2	100	3,544	8.28	No
B.3	100	3,764	6.28	No
B.4	100	7,464	20.15	Yes
B.5	100	2,974	11.10	No
B.6	100	3,842	8.13	No

^a^Replica ID indicates the equilibration output (A or B) followed by the replica number.

^b^This replica was extended until 289 ns and analyzed in this study.

^c^Volume of the water molecules allocated in the periplasmic cavity of MelB (Fig. 1). The volume was calculated with the Volarea plugin^28^ in VMD.
