# Supplementary material for: Helical unwinding and side-chain unlocking unravel the outward open conformation of the melibiose transporter
Source: Sci Rep. 2016 Sep 23;6:33776. doi: 10.1038/srep33776 (PMC5034317; doi:10.1038/srep33776)
Supplement: Supplementary Information [file srep33776-s1.pdf]

Supporting Information File

Helical unwinding and side-chain unlocking unravel the outward open conformation of the melibiose transporter

Li-Ying Wang[1], Vidhya M. Ravi[1], Gérard Leblanc[2], Esteve Padrós[1], Josep Cladera[1,*], and Alex Perálvarez-Marín[1,*]

[1] Unitat de Biofísica, Departament de Bioquímica i de Biologia Molecular, Facultat de Medicina, and Centre d'Estudis en Biofísica, Universitat Autònoma de Barcelona, 08193 Bellaterra, Barcelona, Spain

[2] Direction des Sciences du Vivant, Direction des progammes et valorization, CEA Fontenay-aux-Roses, 92265 Fontenay-aux-Roses CEDEX France

To whom correspondence should be addressed:

E-mail: josep.cladera@uab.cat

alex.peralvarez@uab.cat

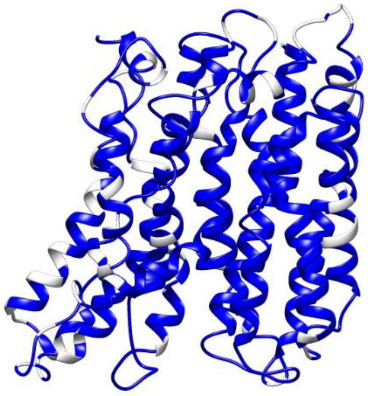

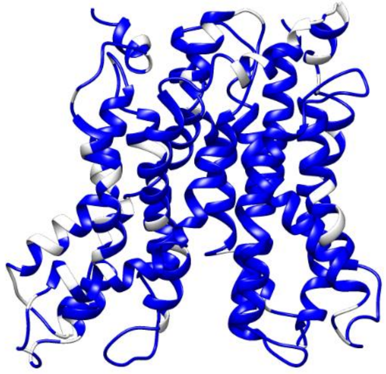

```
sp|P02921|MELB_ECOLI/1-473     1 MSISMTTKLSYGFGAFGKDFAIGIVYMYLMYYYTDVVGLSVGLVGTLFL  49
sp|P30878|MELB_SALTY/1-476     1 MSISLTTKLSYGFGAFGKDFAIGIVYMYLMYYYTDVVGLSVGLVGTLFL  49

sp|P02921|MELB_ECOLI/1-473    50 VARIWDAINDPIMGWIVNATRSRWGKFKPWILIGTLANSVILFLLFSAH  98
sp|P30878|MELB_SALTY/1-476    50 VARIWDAINDPIMGWIVNATRSRWGKFKPWILIGTLTNSLVLFLLFSAH  98

sp|P02921|MELB_ECOLI/1-473    99 LFEGTTQIVFVCVTYILWGMTYTIMDIPFWSLVPTITLDKREREQLVPY 147
sp|P30878|MELB_SALTY/1-476    99 LFEGTAQVVFVCVTYILWGMTYTIMDIPFWSLVPTITLDKREREQLVPF 147

sp|P02921|MELB_ECOLI/1-473   148 PRFFASLAGFVTAGVTLPFVNYVGGGDRGFGFQMFTLVLIAFFIVSTII 196
sp|P30878|MELB_SALTY/1-476   148 PRFFASLAGFVTAGITLPFVSYVGGADRGFGFQMFTLVLIAFFIASTIV 196

sp|P02921|MELB_ECOLI/1-473   197 TLRNVHEVFSSDNQPSAEGSHLTLKAIVALIYKNDQLSCLLGMALAYNV 245
sp|P30878|MELB_SALTY/1-476   197 TLRNVHEVYSSDNGVTAGRPHLTLKTIVGLIYKNDQLSCLLGMALAYNI 245

sp|P02921|MELB_ECOLI/1-473   246 ASNIITGFAIYYFSYVIGDADLFPYYLSYAGAANLVTLVFFPRLVKSLS 294
sp|P30878|MELB_SALTY/1-476   246 ASNIINGFAIYYFTYVIGDADLFPYYLSYAGAANLLTLIVFPRLVKMLS 294

sp|P02921|MELB_ECOLI/1-473   295 RRILWAGASILPVLSCGVLLLMALMSYHNVVLIVIAGILLNVGTALFWV 343
sp|P30878|MELB_SALTY/1-476   295 RRILWAGASVMPVLSCAGLFAMALADIHNAALIVAAGIFLNIGTALFWV 343

sp|P02921|MELB_ECOLI/1-473   344 LQVIMVADIVDYGEYKLHVRCESIAYSVQTMVVKGGSAFAAFFIAVVLG 392
sp|P30878|MELB_SALTY/1-476   344 LQVIMVADTVDYGEFKLNIRCESIAYSVQTMVVKGGSAFAAFFIALVLG 392

sp|P02921|MELB_ECOLI/1-473   393 MIGYVPNVEQSTQALLGMQFIMIALPTLFFMVTLILYFRFYRLNGDTLR 441
sp|P30878|MELB_SALTY/1-476   393 LIGYTPNVAQSAQTLQGMQFIMIVLPVLFFMMTLVLYFRYYRLNGDMLR 441

sp|P02921|MELB_ECOLI/1-473   442 RIQIHLLDKYRKVPP---EPVHADIPVGAVSDVKA               473
sp|P30878|MELB_SALTY/1-476   442 KIQIHLLDKYRKTPPFVEQPDSPAISVVATSDVKA               476
```

Figure S1. 3D structures and sequence alignment for melibiose transporters from *Escherichia coli* (top, corresponding to the equilibrated *E. coli* MelB structure used in this study) and *Salmonella typhimurium* (bottom, 4M64 chain A). The residues shaded in blue indicate identical composition. The sequence identity and similarity between orthologs are 84.87% and 90.12%, respectively [http://imed.med.ucm.es/Tools/sias.html].

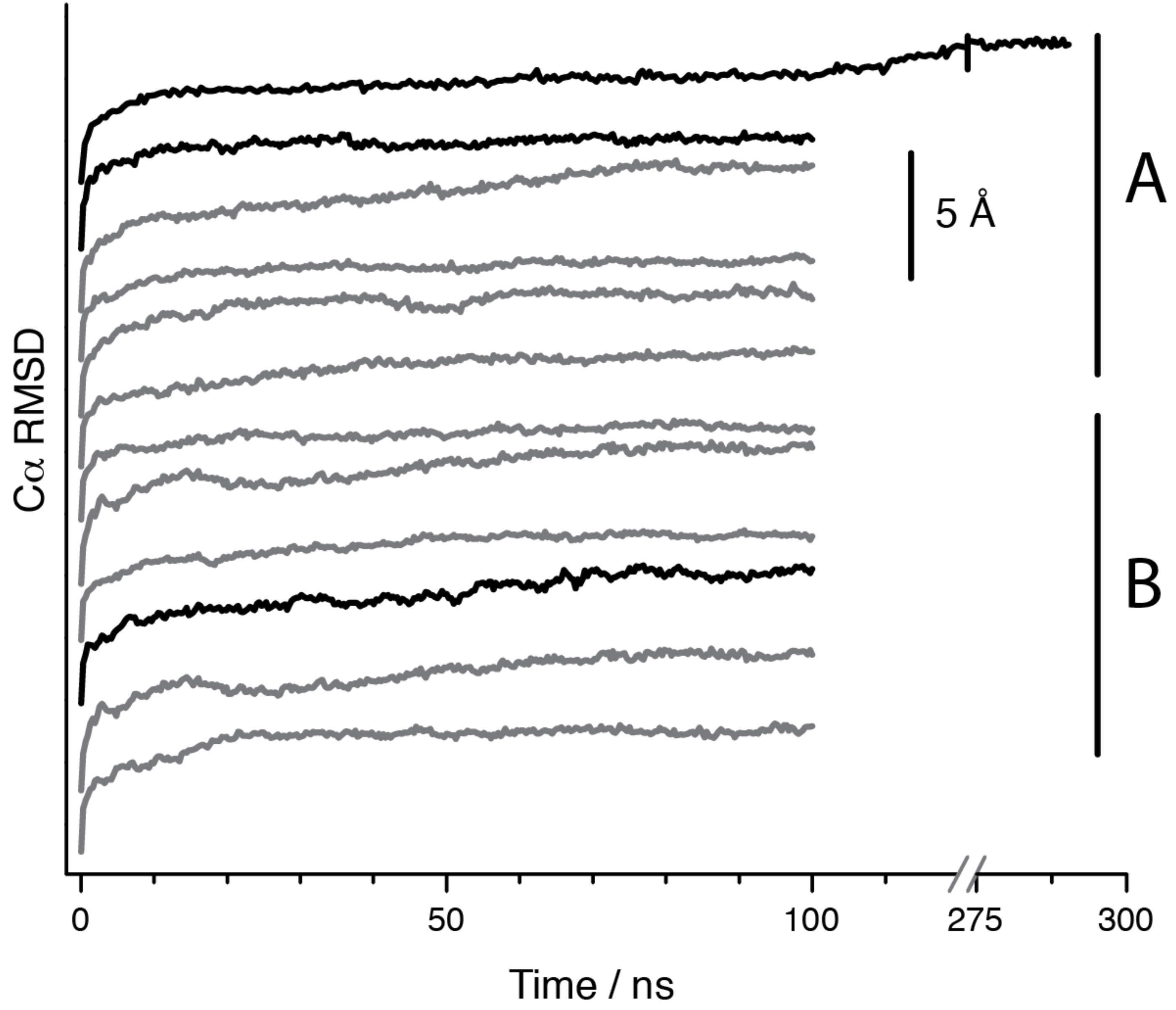

Figure S2. **Cα RMSD for the 100 ns replicas.** Top to Bottom: ordered replicas starting from A.1 until B.6. A and B refer to the equilibration protocol (see Table 1). The replicas resulting into open conformations (Table 1) are indicated in a black solid line.

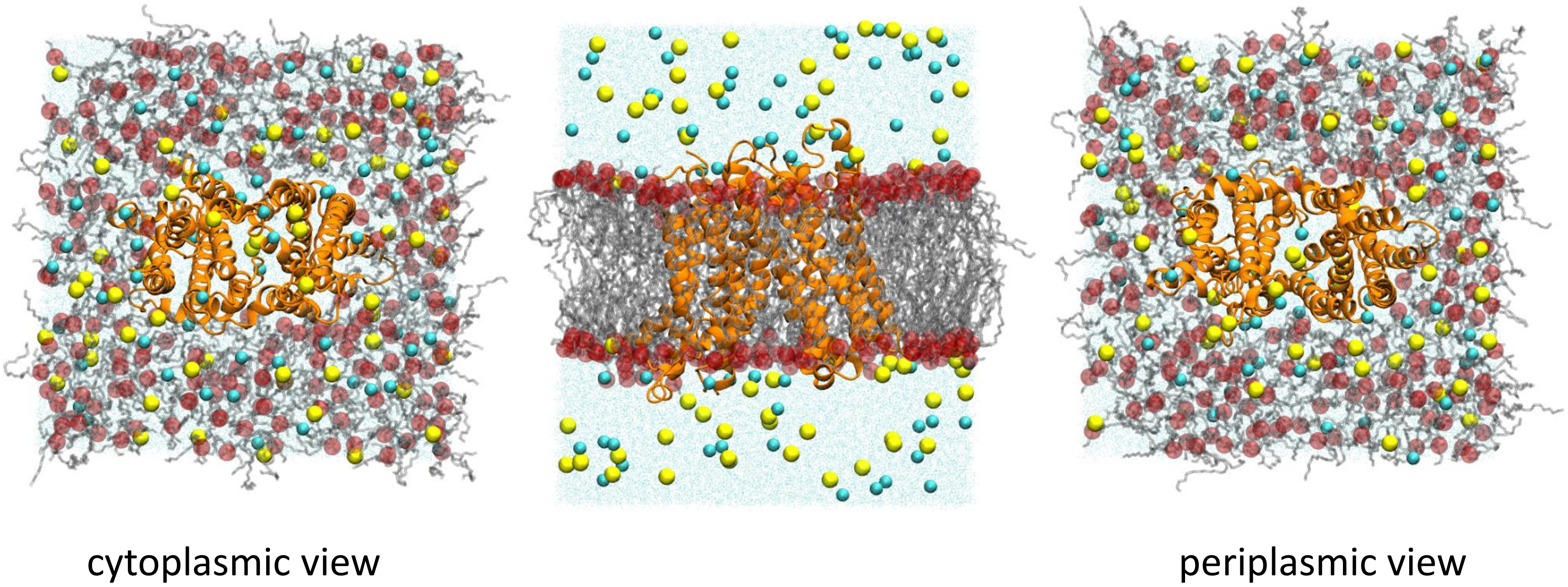

Figure S3. MelB bilayer arrangement of the replica number A.1 at 100 ns snapshot. This replica was extended until 289 ns to analyze the MelB substrate-free fully outward open state. The color code represent in orange a cartoon plot of the protein; grey a bonds plot for the hydrocarbon chain of POPE molècules, with red polar heads; wàters are represented by cyan dots; sodium and chloride are represented by yellow and cyan spheres, respectively.

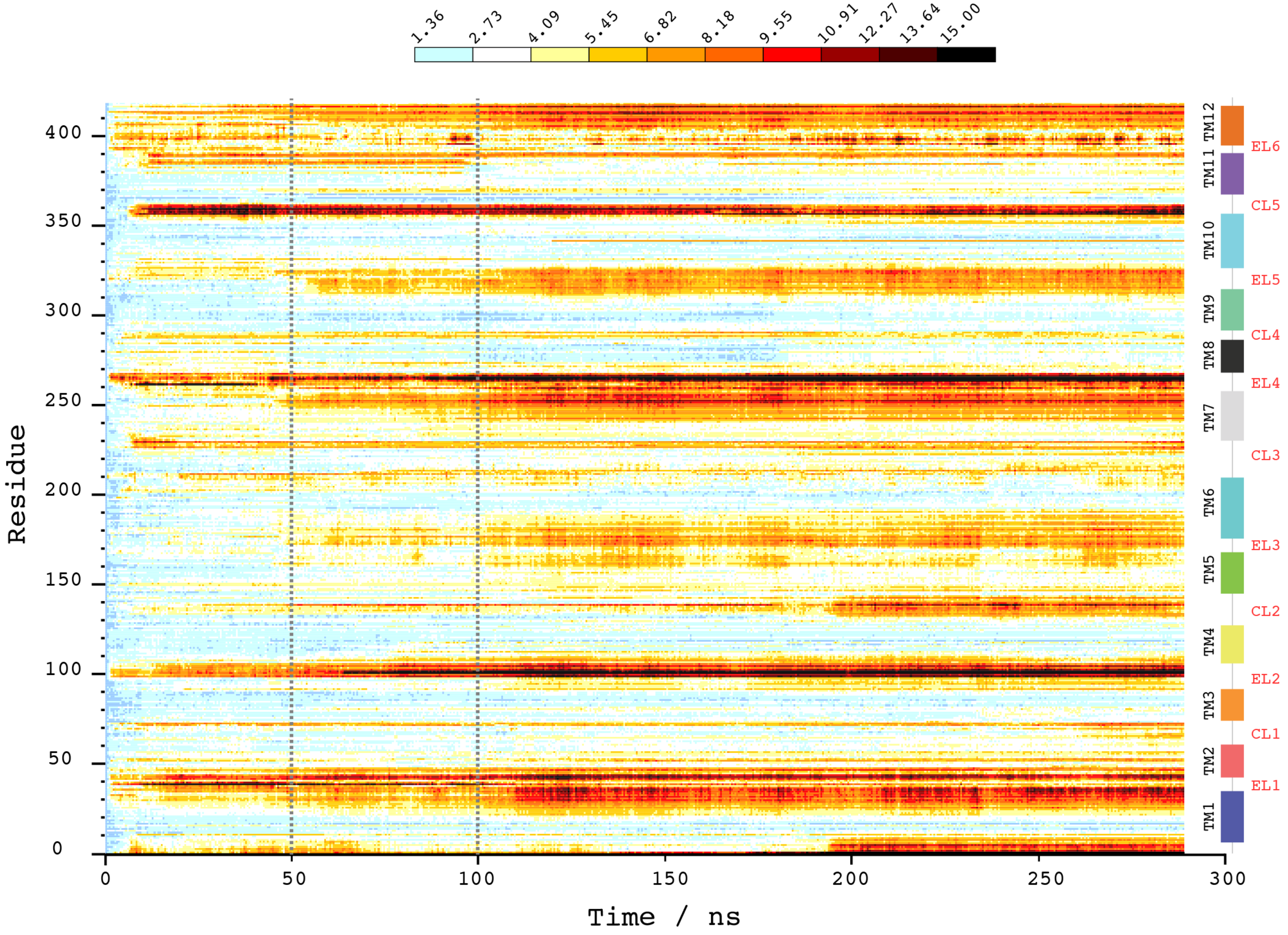

Figure S4. RMSD per residue analysis of the MelB opening. The scale bar indicates RMSD in Å. For clarity we show a cartoon representation of MelB indicating the position of helices and loops. The color code of the TM segments is the same as in Fig. 1.

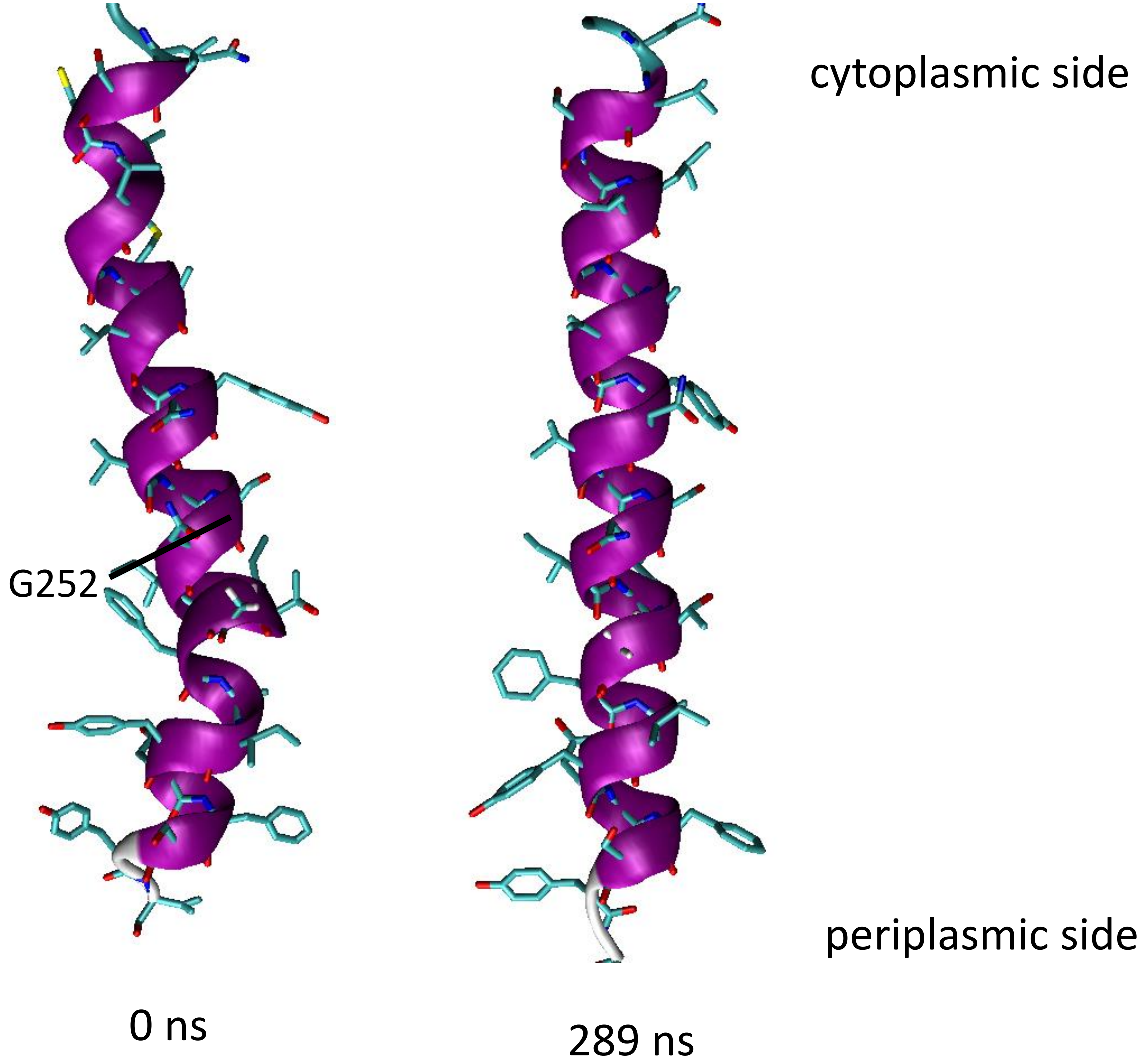

Figure S5. Comparison of TM7 conformation at the beginning and at the end of simulation

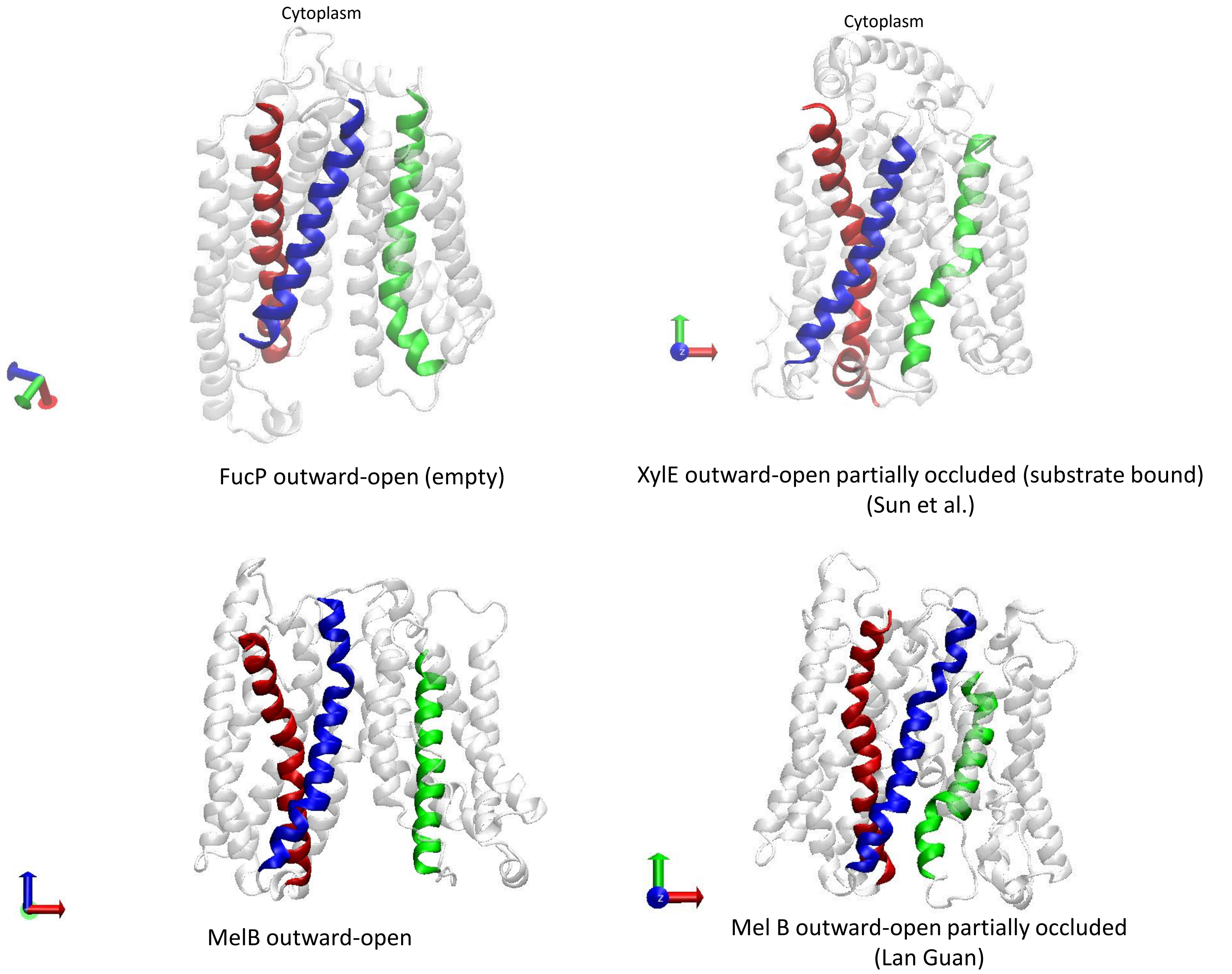

Figure S6. Comparison of TM1, TM5 and TM7 of diferent sugar transporters.

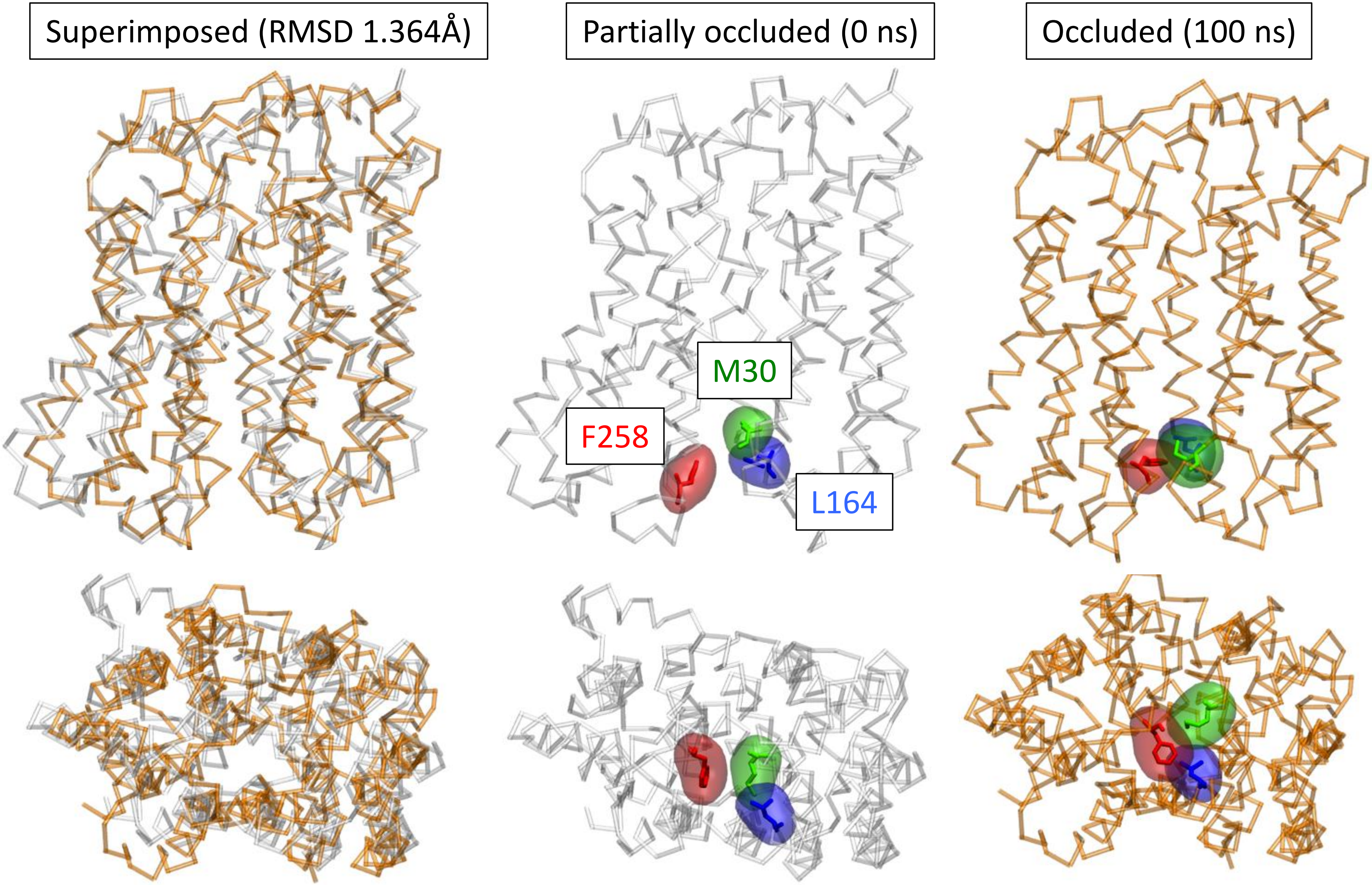

Figure S7. Superimposition of the initial and final state of replica B.1 leading to an occluded state (left, 0 ns and 100 ns depicted as white and orange traces, respectively). Interactions in the periplasmic region of MelB at the beginning (middle, 0 ns) and the end (right, 100 ns) of a simulation leading to an occluded state, replica B.1. Side view (top) and periplasmic view of MelB with residues 30, 164, and 258 depicted as sticks and surface in green, blue, and red, respectively.

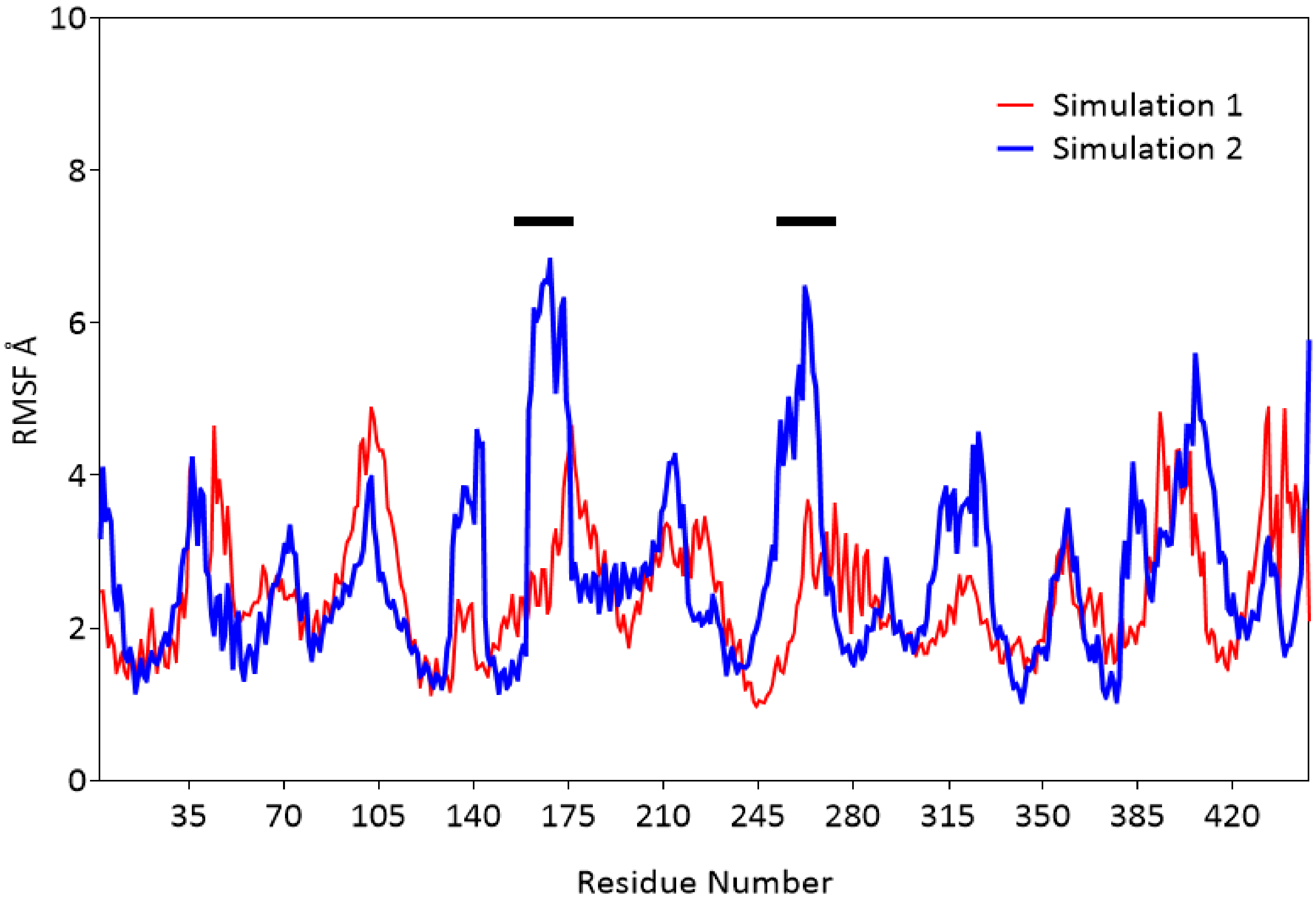

Figure S8. **Cα backbone** RMSF **analysis for simulations yielding occluded (red) and open (blue) states.** The simulation 1 (red plot) shows the very stable fluctuation throughout the dynamics around 4Å while the simulation 2 shows fluctuation in helices 5, 7, and 8 (black bars), indicating a role in opening of the structure.

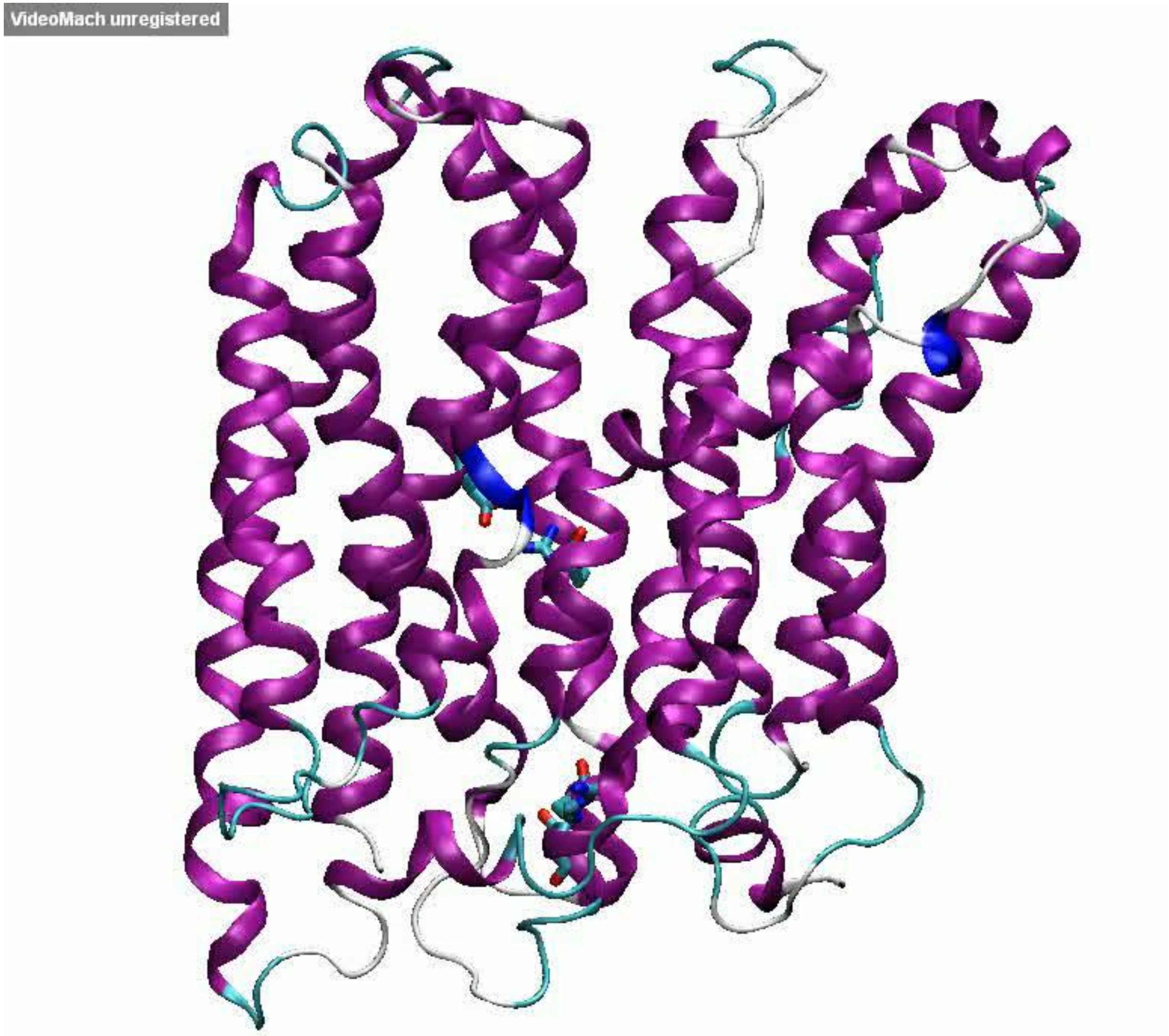

Video S1. **MelB opening.** Opening of melibiose permease during the 289 ns duration of the MD simulation.
